# Night Medical Service

**Published:** 1881-01

**Authors:** 


Night Medical Service.—Now that the new system has been successfully inaugurated in New York, and found to act as well in that city, as in any of the capitols of the Old World, it is sincerely hoped that other, and indeed, all the large cities of this country will see the importance of introducing it into their system of medical service to the sick in sudden emergencies. Why cannot Baltimore step forward, and by introducing the *Night Medical Service* for the benefit of her citizens, add another laurel to her brow, for good works performed?

